# Effects of COVID-19 Lockdown on Otitis Media With Effusion in Children: Future Therapeutic Implications

**DOI:** 10.1177/0194599820987458

**Published:** 2021-11

**Authors:** Mirko Aldè, Federica Di Berardino, Paola Marchisio, Giovanna Cantarella, Umberto Ambrosetti, Dario Consonni, Diego Zanetti

**Affiliations:** 1 Department of Clinical Sciences and Community Health, University of Milan, Milan, Italy; 2 Audiology Unit, Department of Specialist Surgical Sciences, Fondazione IRCCS Ca’ Granda Ospedale Maggiore Policlinico, Milan, Italy; 3 Pediatric Highly Intensive Care Unit, Fondazione IRCCS Ca’ Granda Ospedale Maggiore Policlinico, Milan, Italy; 4 Department of Pathophysiology and Transplantation, University of Milan, Milan, Italy; 5 Otolaryngology Unit, Department of Specialist Surgical Sciences, Fondazione IRCCS Ca’ Granda Ospedale Maggiore Policlinico, Milan, Ital; 6 Epidemiology Unit, Fondazione IRCCS Ca’ Granda Ospedale Maggiore Policlinico, Milan, Italy

**Keywords:** otitis media with effusion, children, COVID-19, lockdown, physical distancing, day care attendance

## Abstract

**Objective:**

To evaluate the role of social isolation during the lockdown due to the SARS-CoV-2 outbreak (severe acute respiratory syndrome coronavirus 2) in modifying the prevalence of otitis media with effusion (OME) and the natural history of chronic OME.

**Study Design:**

Retrospective study.

**Setting:**

Tertiary level referral audiologic center.

**Methods:**

We assessed the prevalence of OME among children aged 6 months to 12 years who attended the outpatient clinic for hearing or vestibular disorders during 2 periods before the lockdown, May-June 2019 (n = 350) and January-February 2020 (n = 366), and the period immediately after the lockdown, May-June 2020 (n = 216). We also compared the disease resolution rates between a subgroup of children with chronic OME (n = 30) who were diagnosed in summer 2019 and reevaluated in May-June 2020 and a similar subgroup (n = 29) assessed in 2018-2019.

**Results:**

The prevalence of OME in this clinic population was 40.6% in May-June 2019, 52.2% in January-February 2020, and 2.3% in May-June 2020. Children with chronic OME had a higher rate of disease resolution in May-June 2020 (93.3%) than those examined in May-June 2019 (20.7%, *P* < .001).

**Conclusion:**

Closure of schools and the physical distancing rules were correlated with a reduction in the prevalence of OME and favored the resolution of its chronic forms among children who attended the outpatient clinic. These data could suggest that in the presence of chronic OME, keeping young children out of group care settings for a period might be beneficial to allow for OME resolution.

Otitis media with effusion (OME), also known as serous or secretory otitis media, is one of the most frequent diseases in childhood. An estimated 80% of all children have had at least 1 episode of OME by the age of 10 years, with a peak of prevalence in the first 2 years of life.^1^ It is characterized by the presence of fluid behind an intact tympanic membrane, without signs and symptoms of acute infection, and it is defined as chronic when the middle ear effusion persists for >3 months.^1,2^

OME is commonly a self-limiting condition, but it can be recurrent and chronic in approximately one-third and one-quarter of affected children, respectively.^2^ Chronic OME is potentially associated with conductive hearing loss and middle ear complications, often leading to speech and behavioral problems and poor school performance.^2,3^ It represents the most common cause of hearing impairment in children in the developed world,^4^ negatively affecting quality of life.

The high prevalence of OME in young children has been associated with the anatomic and functional immaturity of the eustachian tube,^4^ the higher rate of upper respiratory tract infections (URTIs) due to exposure to viruses and bacteria in day care centers,^2,5^ and the often concomitant hypertrophy of the adenoid tissue.^6^ The forced isolation of children at home during the recent SARS-CoV-2 pandemic (severe acute respiratory syndrome coronavirus 2) has provided an opportunity to verify the impact of nonattendance at day care centers and schools on the prevalence of OME.

The aims of the present study were to evaluate the prevalence of OME before and after the national lockdown on all activities and to determine the influence of social isolation on the natural history of chronic OME in children who attended the pediatric outpatient audiology clinic.

## Materials and Methods

The present retrospective study included all children aged 6 months to 12 years who attended the pediatric outpatient audiology clinic in the Fondazione IRCCS Ca’ Granda, Ospedale Maggiore Policlinico (Milan, Italy), as a first or follow-up visit for hearing, speech, language, or vestibular disorders. The study exclusion criteria were as follows:

- Age <6 months or >12 years
- Otomicroscopic evidence of tympanosclerosis, cholesteatoma, eardrum perforation, or complete stenosis or atresia of the external auditory canal
- Craniofacial anomalies, cleft palate, or syndromes characterized by anatomic and functional impairment of the eustachian tube
- A recent history of medical treatment (<2 months before visit), such as antibiotics, steroids, or other medications or interventions that could have transiently cleared the OME
- Contraindications to tympanometry—otitis externa, acute otitis media, otorrhea, recent ear surgery (eg, myringoplasty, tympanoplasty, and stapedectomy), presence of tympanostomy tubes, foreign body in the external auditory canal

All children underwent otomicroscopy with earwax removal (if necessary), tympanometry (compliance, ear canal volume, and middle ear pressure measurements), pure tone or behavioral audiometry (depending on age), and, in selected cases, threshold detection by air conduction (AC) and bone conduction (BC) auditory brainstem responses.

The prevalence of OME was investigated in this clinic population 3 times—the first 2 before the lockdown due to coronavirus disease 2019 (COVID-19) and the last immediately after relaxation of the tight restriction measures. The 3 periods were as follows: May 1–June 30, 2019 (period 1); January 1–February 29, 2020 (period 2); and May 1–June 30, 2020 (period 3). The prevalence of OME among children who visited during period 1 was compared with that among children who were examined during periods 2 and 3. We then selected a subgroup (subgroup A) that included all children with chronic OME who were

- Diagnosed at our clinic during June 1–August 30, 2019
- Reexamined at our clinic during December 1, 2019–February 29, 2020, finding that OME had not resolved
- Reevaluated at our clinic during period 3

We also selected a corresponding subgroup (subgroup B) of children with chronic OME who were

- Diagnosed at our clinic during June 1–August 30, 2018
- Reexamined at our clinic during December 1, 2018–February 28, 2019, finding that OME had not resolved
- Reevaluated at our clinic during period 1

We assessed the disease resolution rate of subgroups A and B during periods 3 and 1, respectively, and then compared the corresponding results.

OME was confirmed if all the following criteria were fulfilled: type B tympanogram (flat); otomicroscopic evidence of middle ear effusion, defined by a yellowish retracted tympanic membrane and by air-fluid level or bubbles in the middle ear; and mild to moderate conductive hearing loss.

Tympanograms were obtained with a standard 226-Hz probe tone and classified as follows:

*Type A*: normal compliance and middle ear pressure
*Type B*: low compliance with no discernible peak
*Type C*: normal compliance with negative middle ear pressure, often associated with a retracted tympanic membrane due to eustachian tube dysfunction and divided into 2 subtypes^7^: (type C1) if pressure is from −100 to −199 mm H_2_O and (type C2) if pressure is from −200 to −400 mm H_2_O

AC and BC pure tone audiometry or behavioral audiometry with AC and BC auditory brainstem responses were used to assess the typical air-bone gap of conductive hearing loss. The criteria to define OME resolution were as follows: change from type B to type A tympanogram, complete recovery of the conductive hearing loss, and normal aspect of the eardrum on otomicroscopy with no more evidence of middle ear effusion.

The study was conducted according to the World Medical Association’s Declaration of Helsinki and approved by the ethical committee (Area 2 Milano) of the Fondazione IRCCS Ca’Granda Ospedale Maggiore Policlinico (370_2020). Informed consent was obtained by the children’s parents.

### Statistical Analysis

The units of analysis were children, and we considered the worse ear. We compared the children’s sex and age characteristics and the distribution of the types of tympanograms in the 3 periods, using chi-square tests and multivariable logistic regression models adjusted for sex and age category. To assess whether there was an effect on the prevalence of tympanogram B according to sex and age over time, we fitted multivariable logistic regression models containing time × sex and time × age class product terms to conduct Wald tests and calculate *P* values for these interactions. We analyzed the proportions of normalization (type B to type A tympanograms) in May-June 2020 and May-June 2019 using Poisson regression models with robust variance, and we assessed effect modification by sex by inserting a period × sex product term. Statistical analyses were performed with Stata version 16 (StataCorp).

## Results

A total of 932 children with hearing, speech, language, or vestibular disorders were evaluated, including children affected by metabolic and genetic disorders who needed an audiologic assessment. A total of 350, 366, and 216 children were assessed during periods 1, 2, and 3, respectively (**Table 1**).

**Table 1.** Characteristics of the Children in the 3 Periods.

	1: May-Jun 2019		2: Jan-Feb 2020		3: May-Jun 2020		*P* value^*a*^	
Variable	No.	%	No.	%	No.	%	2 vs 1	3 vs 1
Sex								
Male	216	61.7	226	61.7	138	63.9	.99	.60
Female	134	38.3	140	38.3	78	36.1		
Age, y								
<3	116	33.1	124	33.9	92	42.6	.88	.02
3 to <7	154	44.0	164	44.8	70	32.4		
≥7	80	22.9	78	21.3	54	25.0		
All	350	100.0	366	100.0	216	100.0		

a Chi-square test.

**Figure 1.**
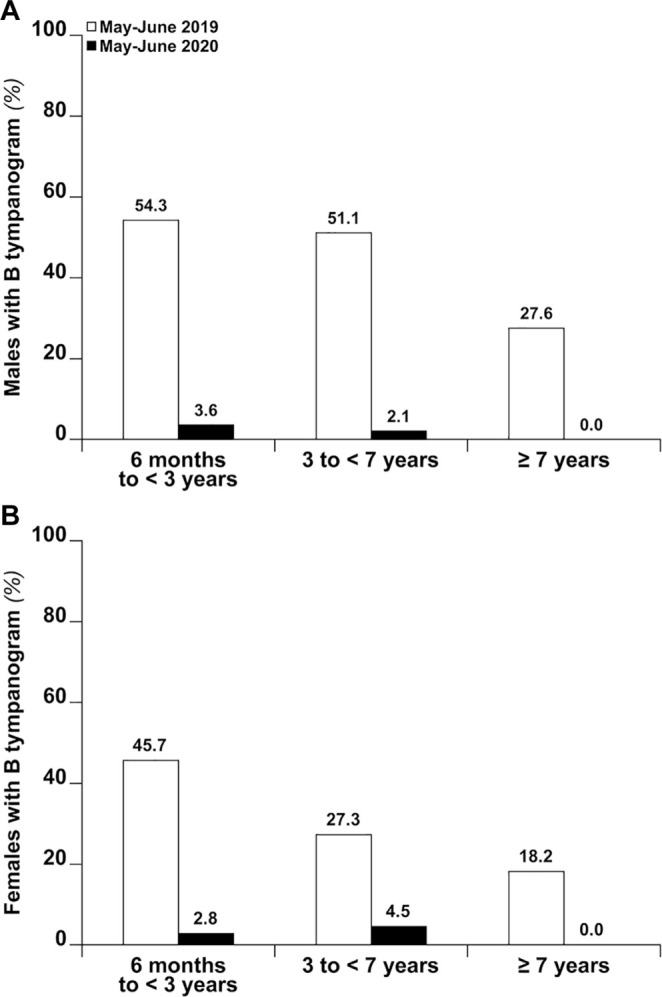
Prevalence of type B tympanograms by age between (A) males and (B) females, comparing May-June 2019 and May-June 2020.

A higher prevalence of males than females was observed, with no sex differences among the 3 periods (**Table 1**). Similarly, there were no age class differences between the patients examined in periods 1 and 2, while in period 3, a higher frequency of younger children (<3 years) attended our department. The prevalence of type B tympanograms was 40.6% in the first period; it increased to 52.2% in the second period and then dropped to 2.3% in the third (**Table 2**). The prevalence difference between periods 3 and 1 was −38.3% (95% CI, –43.8% to –32.7%). The reduction in the prevalence of type B tympanograms in period 3 was similar across sex (interaction, *P* = .39) and age (interaction, *P* = .78; **Figure 1**).

**Table 2.** Tympanograms for the 3 Periods.

	1: May-Jun 2019		2: Jan-Feb 2020^*a*^		3: May-Jun 2020^*b*^	
Tympanogram	No.	%	No.	%	No.	%
A	157	44.9	98	26.8	204	94.4
B	142	40.6	191	52.2	5	2.3
C1	31	8.9	39	10.7	6	2.8
C2	20	5.7	38	10.4	1	0.5
Total	350	100.0	366	100.0	216	100.0

a Period 2 vs 1: chi-square, *P* < .001.

b Period 3 vs 1: chi-square, *P* < .001.

**Figure 2.**
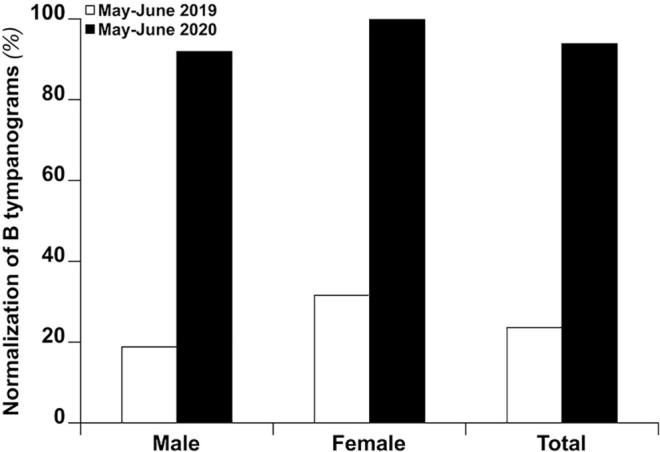
Normalization of type B tympanograms in the 2 subgroups of children who were diagnosed with chronic otitis media with effusion, comparing May-June 2019 and May-June 2020.

A clear age effect was also observed at all periods of the study (**Figure 1**). Concerning the subgroups of children with chronic OME, subgroup A included 30 children (23 male and 7 female), with an average age of 4.7 years, while subgroup B included 29 children (18 male and 11 female), with an average age of 5.0 years. At the May-June 2020 assessment, the children belonging to subgroup A presented a greater rate of normalization of tympanograms (93.3%, 28/30) from type B to type A than the children belonging to subgroup B, who were evaluated May-June 2019 (20.7%, 6/29, *P* < .001; **Figure 2**). No sex differences in resolution rates were recorded: 91.3% (21/23) in males and 100% (7/7) among females (interaction, *P* = .58).

## Discussion

A nationwide lockdown was imposed by the Italian government from March 9 to May 18, 2020, to contain the COVID-19 outbreak. All schools and day care centers were completely closed, and children were strictly forbidden to leave their homes. The restrictive measures adopted by the authorities represent a unique and exceptional event in recent world history and provided a great opportunity to evaluate the impact of social isolation on OME. The prevalence of OME in children referred to our outpatient audiology clinic during the 2-month periods before the COVID-19 lockdown (periods 1 and 2) was higher than what is usually reported in the literature.^2,8^

These findings could be due to several reasons. First, our clinic is a tertiary-level referral audiologic center to which patients from other departments, such as pediatrics, otolaryngology, infectious disease, and neuropsychiatry, are sent to be evaluated for suspected hypoacusis, severe middle ear diseases, and language delay. Second, the metropolitan city of Milan is one of the most polluted areas in Europe,^9^ and this probably implies a greater risk of OME as compared with other Italian regions.^10,11^

Seasonal variations in the prevalence of OME, with a peak in winter that is related to the increased incidence of URTIs,^12-14^ are confirmed by our finding of a higher rate of affected children during period 2 versus period 1. In agreement with previous studies,^4,8^ we found a decreasing prevalence of OME with age, reflecting the progressive maturation of the immune system and changes in the anatomic orientation, size, and shape of the eustachian tube, irrespective of the period of analysis. We also confirmed a higher prevalence of OME in males for each age group and during both periods of observation.^2,15^ The reasons for this sex difference are still unknown, but possible hypotheses include genetic determinants of susceptibility to OME,^16^ defective pneumatization of the mastoid process,^17^ and different impacts of sex hormones on Th1/Th2 cytokine balance (T helpers 1 and 2).^18^

Audiologic evaluations performed after the loosening of the strict 2-month lockdown due to COVID-19 demonstrated that the prevalence of OME drastically decreased, shifting from 40.6% in period 1 to 2.3% during period 3 (–38.3%). This remarkable decrease in the prevalence of OME was detected in each age group and in both sexes. It might be assumed that this drop, so stunning in size, could be related to a decrease in the overall number of patients affected by serious clinical conditions who were referred to our outpatient clinic after the lockdown period. This was not the case because when the office was reopened for visits, adequate measures of social distancing were adopted in the hospital waiting room, and the visit hours were extended, allowing maintenance of the previous appointment schedule and giving even more priority to younger otitis-prone children (<3 years).

One could also argue about the definition of and diagnostic criteria for OME. Although tympanometry is believed to be a fairly reliable technique in diagnosing OME, some false positives may occur.^19^ For this reason, we purposefully included in the study group only children showing all 3 diagnostic criteria for OME (type B tympanogram, positive otomicroscopic findings, and ipsilateral conductive hearing loss) to reduce a possible diagnostic bias.

In the present study, the follow-up visits for children with chronic OME who were diagnosed in summer 2019 and checked again in May-June 2020 revealed a much higher rate of complete resolution of the disease (93.3%) as compared with a homogeneous cohort diagnosed in summer 2018 and reassessed in May-June 2019 (20.7%). This trend was significant in males and females and for all age groups, highlighting the positive effects of social isolation on OME in children of all ages.

The obligated avoidance of interpersonal contacts and rigorous respect of the hygienic-behavioral rules appear to have significantly contributed to containing the spread of not only COVID-19 but also all other infectious diseases that underlie the development or persistence of OME, such as URTIs.^20-23^ Children attending day care centers are more frequently exposed to resistant organisms, whose transmission and aggressiveness are favored by class sizes that are large, increased peer-to-peer close contact, and indiscriminate use of antibiotics.^24,25^ Moreover, psychological stress in sick children who attend day care centers leads to higher cortisol levels, especially in children <36 months old, with consequent alteration of the immune response and an increased risk of OME.^26,27^

In our opinion, the results of our study could have important implications for clinical practice, suggesting that keeping children at home for a period as short as 2 months might allow for the resolution of most cases of severe and resistant OME. This type of approach, even if potentially beneficial for all age groups, might be especially considered for young children, who are the most prone to otitis. Among the shortcomings of this solution is that in most circumstances, the availability of a caretaker other than a parent (eg, babysitter) may be burdensome or unavailable to working parents.

Despite the importance of the major findings, the study has several limitations. First, it is retrospective and not population based. A limited number of patients were assessed in a single audiologic center. Furthermore, there are a few sources of bias, including the possibility that some patients postponed or sought treatment elsewhere, as well as a reasonable reluctance of parents to take their children back to the hospital for a follow-up visit due to the COVID-19 outbreak.

Even during the temporary interruption of day care center attendance, additional risk factors, such as allergies or interactions with older siblings and parents, could predispose to URTIs and should be considered in future studies. Although it has been reported that children with asthma and allergic rhinitis are generally more likely to develop URTIs,^28^ the effects of the lockdown on allergic population are still debated and unclear.^29,30^

Currently, a new partial lockdown due to COVID-19 outbreak has been adopted in Italy, with the closure of all shops and restaurants, allowing only children aged <12 years to attend school. As soon as the new strict measures are relaxed, it will be interesting to reevaluate these patients, to understand if the use of face masks and the limitation of social activities are sufficient alone to reduce the prevalence of OME, regardless of day care center and school attendance.

Future developments of this research could involve (1) periodic reevaluation of children who experienced a resolution of OME after the lockdown, (2) comparison of our data with those of a country in which no social isolation due to COVID-19 outbreak was performed, and (3) proposal for a national register-based study of otitis media.

## Conclusions

The drastic measures taken by the Italian government to contain the spread of COVID-19 have incidentally had a positive impact on OME, from a preventive and therapeutic point of view, among children who attended our pediatric outpatient audiology clinic.

To our knowledge, the present study represents the first report on the effects of social restriction on OME in children examined directly at the hospital in the first 2 months after the lockdown. Our findings highlighted a lower prevalence of OME among the pediatric patients referred to our hospital and a higher rate of resolution of the chronic forms of OME as compared with a similar period 1 year before the COVID-19 outbreak.

Further studies on the general pediatric population are necessary to determine if interrupting day care center attendance for a brief period could be a viable alternative to medical or surgical therapies in the treatment of severe and resistant cases of OME in children.
